# The Impact of Technological Progress and Climate Change on Food Crop Production: Evidence from Sichuan—China

**DOI:** 10.3390/ijerph19169863

**Published:** 2022-08-10

**Authors:** Abbas Ali Chandio, Yasir A. Nasereldin, Dao Le Trang Anh, Yashuang Tang, Ghulam Raza Sargani, Huaquan Zhang

**Affiliations:** 1 College of Economics, Sichuan Agricultural University, Chengdu 611130, China; 2 College of Economics and Management, Nanjing Agricultural University, Nanjing 210095, China; 3 Department of Agricultural Economics & Agribusiness, Faculty of Natural Resources & Environmental Studies, Kordofan University, EI Obied 51111, Sudan; 4 Faculty of Business and Economics, Phenikaa University, Hanoi 100000, Vietnam

**Keywords:** global warming, technological advancement, staple crop, GMM model

## Abstract

Agriculture is an integral sector in China mandated to feed over 1.3 billion of its people and provide essential inputs for many industries. Sichuan, a central grain-producing province in Southwest China, is a significant supplier of cereals in the country. Yet, it is likely to be threatened by yield damages induced by climate change. Therefore, this study examines the effects of technological progress (via fertilizers usage and mechanization) and climatic changes (via temperature and precipitation) on the productivity of main food crops, such as rice (*Oryza sativa*), wheat (*Triticum aestivum*), and maize (*Zea mays*) in Sichuan province. We employ the generalized method of moments (*GMM*) model to analyze Sichuan provincial data from 1980 to 2018. Our findings show a positive nexus between fertilizers use and yields of main food crops. Only rice and maize yields are significantly improved by mechanization. Increased average temperature reduces rice and wheat yields significantly. Rainfall is unlikely to have a significant impact on agricultural production. The study suggests that the Chinese government should consider revising its strategies and policies to reduce the impact of climate change on food crop production and increase farmers’ adaptive ability.

## 1. Introduction

Food security has been jeopardized by several linked variables, including population increase, environmental issues, and land degradation. It is predicted that the world’s population will reach around 10 billion by 2100 [1]. Indeed, China is the world’s most populated nation, feeding one-fifth of the world’s people while utilizing just 8% of the world’s agricultural land [2]. The Chinese government’s long-term aim is to provide foods for the country’s rapidly rising population. Furthermore, food consumption will continue to rise due to population expansion and economic development. However, arable land and other productive resources will diminish due to climate change’s impact on agricultural production [3,4]. For China to achieve food security by 2030, China’s grain production must rise by an estimated 142% while having just 85% of its existing cropland [5]. Hence, technical progress is essential for sustainable agricultural production in the country.

Technology has long caught the attention of economists and economic historians as the key to long-term economic development. Contemporary economic growth theory demonstrates that technological progress should be the primary driver of long-term economic growth. This means that China’s agricultural development should be driven by advances in technical change/progress rather than traditional factor input growth, which are the government’s primary aims [6]. Over the last three decades, advancements in agricultural technology have been the primary driving factor behind increases in wheat, rice, and corn yields in major crop-producing nations [7]. A significant contribution to increasing food crop yields in China has come from the rapid development of agricultural biochemistry technology, mechanical technology, and cultivation technology.

In addition, chemical fertilizers and large agricultural machinery are used in the construction of irrigation and water conservation facilities, which have been integral in increasing crop yields. Nonetheless, several hurdles to boosting agrarian productivity to fulfill this need include increased food consumption and land and water resources [8]. A study by Huang et al. [9] predicts that China’s total food self-sufficiency will likely decline from 94.5 percent in 2015 to roughly 91 percent by 2025.

Climate change is projected to exacerbate China’s food security concerns. China’s yearly average temperature has been steadily increasing over the last six decades, and the trend is expected to continue [10]. According to scientific studies and observation data, climate change has substantially influenced China’s agriculture and crop yields, such as wheat, maize, and rice [11]. It is well acknowledged that the primary mechanism of climate change influencing China’s agriculture is the increase in temperature and the increase in variability of precipitation [12].

A growing body of research indicates that crop yields in arid and semi-arid areas are disproportionately impacted by climate change, notably via aridification, which severely hinders agricultural expansion in these places [13]. With a total planting area of 30,190 hectares, China produced 148.5 million tons of cereals in 2018, making it the world’s biggest cereal producer and accounting for around 28% of global production [14]. With 20–36 percent in the next 20–80 years, its grain yield is predictable to decrease because of climate change [15]. The unusual changes in temperature and rainfall can slow down the growth of food crops, resulting in a drop in the average yield of grains [16]. 

Numerous studies have shown that climate change dramatically lowers the production potential of various crops at various sizes (i.e., global, regional, and local scales). For example, between 1980 and 2008, the worldwide production potentials of wheat and maize were lowered by around 6% and 4%, respectively. Climate change enhanced the Fertile Crescent’s wheat production potential in Asia [17] and decreased rice and wheat production potential in the upper Indian Ganga Basin [18]. In China, output potential was lowered in the Northeastern region by 6.45 percent due to a hotter environment and less precipitation [18].

**Figure 1:**
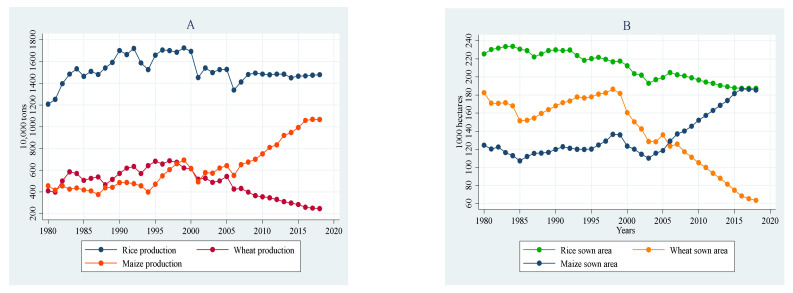
Production (**A**) and sown area (**B**) of food crops in Sichuan province. (Source: China Rural Statistical Yearbook of Sichuan Province [20]).

Sichuan province is the leading agricultural province and the only central grain-producing province in Southwest China. In 2018, the grain production of Sichuan province reached 34.937 million tons, including 14.786 million tons of cereal, 2.473 million tons of wheat, and 10.663 million tons of corn [19]. The trends of food crop production and the sown area are shown in Figure 1. Cereals are the most significant food in the province, independent of the land under cultivation or the total amount of grain produced. Recently, the area where cereals are grown has stabilized. However, the area planted with wheat has dropped by almost half in the last decade, and the area planted with corn has fallen by nearly half in the same time. The climatic condition has also changed, typically exhibiting higher temperature characteristics and less precipitation. This is also induced by the rise in CO_2_ emissions reported in the province annually [2].

**Figure 2:**
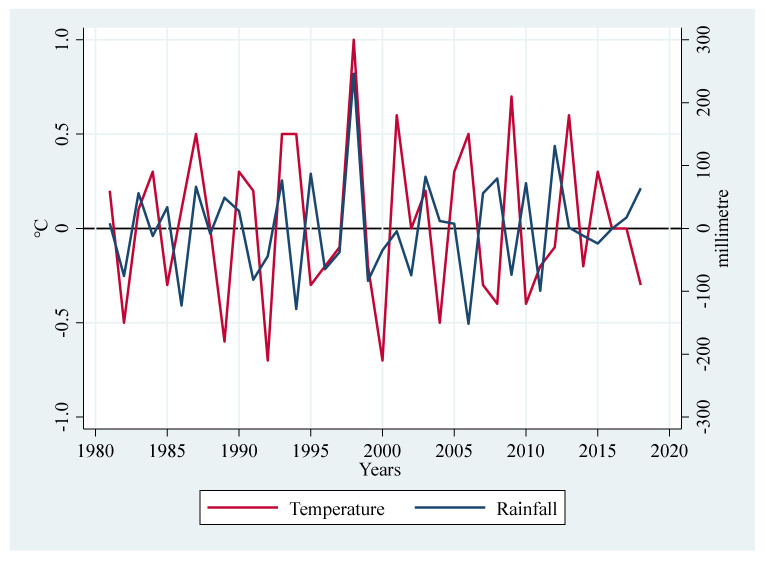
Temperature change and rainfall change in Sichuan province (Source: Weather Bureau of Sichuan Province [22]).

Changes in temperature and rainfall are important indicators that show how the climate changes. In the last 50 years, from 1960 to 2013, the surface air temperature in China has risen by 0.27 °C every 10 years. This is faster than the rate of warming in the rest of the world and in the northern hemisphere over the same period. Also, the average precipitation that falls annually across the country is rising [21]. Most provinces in China have trouble growing food crops because of changes in temperature and rainfall. This causes the average grain yield to go down [1]. Sichuan province’s average temperature in 2019 was 15.4 °C, which is 0.5 °C higher than the average temperature for the year. This is the ninth highest average temperature in history. At the same time, it rains less during the year, which shows that rain unevenly falls in Sichuan province [1]. Figure 2 shows the trends of temperature and rainfall changes in Sichuan province from 1980–2018. 

The present paper explores how technical factors (i.e., fertilizers use and mechanization) and climate change (via temperature and rainfall) impact the production of staple food crops (i.e., rice, wheat, and corn) in Sichuan province by using the GMM approach.

## 2. Review of Literature

In this study, we developed the literature review based on two sub-sections: technological progress and food crops production nexus and climate change and food crops production nexus.

### 2.1. Technical Progress and Food Crops Production Nexus

Technical progress in agriculture necessitates a fresh look at agricultural production concerns, especially in recent years. Fixed technological assets that enable one to substitute human labor with objectified labor define the degree of agricultural production technification (mostly tractors, machines, and tools). Technification in sustainable development also includes lowering environmental hazards by boosting productivity, reducing the material intensity and energy consumption, and lowering the failure rate of machinery and equipment [23]. 

There are two types of agricultural technology: mechanical and biochemical. Mechanical technology saves labor, whereas biochemical technology improves crop yields per unit area, known as the *‘induced innovation theory’* [23]. The *‘Induced innovation hypothesis’* has been examined in China’s agriculture and discovered that under the combined constraints of soil scarcity and growing labor costs, Chinese agriculture employs machines to substitute labor and a significant quantity of fertilizer to make up for soil limitations [24]. Because of this, from 1978 to 2016, China’s fertilizer consumption climbed over six times, accounting for more than a third of worldwide fertilizer usage. Yet, this period’s utilization rate was less than half of the world average [23].

The agricultural sector relies heavily on fertilizers, referred to as ‘food’ for crops. Fertilizer is becoming more crucial in China’s agricultural productivity due to resource scarcity, shrinking arable area, and a rising population [25,26]. On the other hand, a different perspective on agricultural development, based on the literature on the green revolution, views mechanization as a critical component in increasing productivity through fertilization, improved seeds, and irrigation [27]. Smallholder farmers have been pushed to participate in modern agricultural production in China via the development of agrarian mechanization services. 

Since 2004, agricultural mechanization services in rural China have grown significantly [28]. Technological innovation is becoming increasingly important as China moves from high-speed economic growth to high-quality growth. It is essential to measure the level of technological progress in China and look into how it relates to the production of the leading food crops. Thus, this section highlights the related empirical studies that employed several modeling approaches to evaluate the scope. 

As shown in Table 1, Chandio et al. [29] (Table 1), for example, concluded that adopting agricultural technology, such as fertilizer usage, has favorable long and short impacts on rice output in Nepal. In another study, Chandio et al. [3] studied the influence of global warming on China’s agricultural production from 1982 to 2014; the study reported that fertilizer usage was positively and substantially related to agricultural output in the long- and short-term analyses. In another recent work, Chandio et al. [30] analyzed the long- and short-term implications of climate change on maize output in Nepal. They discovered that fertilizer usage considerably impacted maize yield at both times. Moreover, Chandio et al. [31] utilize data from 1977 to 2014 to investigate the effect of global climate change and technological progress on grain output in Pakistan. It has been shown that using fertilizers has a considerable beneficial impact on grain output in the short and long run.

More recently, in the case of Thailand, Chandio et al. [32] utilized data from 1969 to 2016 to analyze the influence of climatic change and financial growth on rice production. Using organic fertilizers considerably improved rice farming in the long and short term. Similarly, Gul et al. [33] have also indicated a significant positive effect of fertilizers consumption on primary food crop production in Pakistan. In a more recent paper, Gul et al. [34] investigated the impact of meteorological and non-meteorological change variables on rice output by using yearly data from 1970 to 2018. They observed that fertilizers consumption has a favorable and significant long-term influence on rice yield, whereas agricultural machinery had a negative effect on rice output in Pakistan. Likewise, Chandio et al. [35] concluded that the usage of fertilizers had a positive influence on the rice yield in the long run, but it had a negative effect on the productivity of rice crops in the short run.

In the case of Pakistan, Rehman et al. [36] reported a positive and significant linkage between output fertilizer usage and agricultural output. Pickson et al. [37] used data from 1998 to 2017 to investigate the influence of climate change on rice production in China. They discovered that fertilizer use had a favorable long-run and substantial impact on rice output. In the context of Pakistan, Ali et al. [38] studied the effect of climatic factors and technological progress on major food crops. They concluded that fertilizer use increases wheat yields significantly but has no effect on rice output. Furthermore, agricultural machinery has a positive but insignificant impact on essential food crops. Ali et al. [39] recently discovered in another study that fertilizer use has a considerably favorable long-run influence on sugarcane crop production; on the other hand, agricultural machinery has an adverse and significant effect on sugarcane crop yield in Pakistan. 

### 2.2. Climate Change and Food Crops Production Nexus

Both developing and developed nations face significant challenges due to global warming. Agriculture is one of several economic sectors that has already been negatively affected by climate change [40]. Climate change, irregular rainfall, and a rise in the severity of droughts and floods have a more significant effect on agriculture than on other industries [41]. Rice, wheat, and maize are the most usually cultivated and eaten crops globally. However, climate change has a considerable impact on the production of grain crops [42]. Because of the struggle between population expansion and food production, altering climatic circumstances might lead to significant food insecurity [43,44].

Non-governmental organizations, international organizations, and world leaders have taken notice of climate change as a worldwide issue in recent years, even though it has become a global concern [45]. As a result of climate change, emerging economies, particularly those relying on agriculture, have been more affected than established ones [40]. This is partly due to the industrialized nations’ capacity to adapt swiftly and effectively to climatic disasters and ameliorate their detrimental consequences [45]. As a result, the scientific and economic literature on the effects of climate change has placed a premium on the agricultural sector in emerging nations. Thus, considerable empirical research has calculated the impact of climatic factors on agricultural productivity in various locations and shown that climate change has a diverse influence on agricultural output.

From 1985 to 2016, Warsame et al. [45] studied the impact of climate change on Somalia’s agricultural production (see Table 2). It was observed that rainfall increases agricultural output in the long run but decreases it in the short run. At the same time, temperature negatively influences crop productivity both long and short term. Similarly, Ul-Haq et al. [41] studied the effect of climatic characteristics on agricultural productivity in South Asia’s emerging areas, arguing that rainfall is inversely related to agricultural production. Temperature, on the other hand, is positively correlated with agricultural productivity.

In the case of Pakistan, Ali et al. [38] evaluated the influence of climatic change on sugarcane production between 1989 and 2015. They observed that rainfall considerably influences sugarcane output, but the temperature has a small and favorable effect in the short term. Gul et al. [33] used annual data from 1985 to 2016 to investigate the climate influences on essential food crop yields in Pakistan. Overall, they concluded that although average temperatures have a negative impact on food production, in the long run, average rainfall has a positive impact. 

Using yearly series data from 1970 to 2018, Gul et al. [34] analyzed the impact of climatic factors on Pakistan’s rice yield. They determined that increased temperature has a negative influence on rice yield. Rice crop yields in Pakistan were shown to benefit both in the long and short term from higher temperatures, according to Chandio et al. [35]. Ozdemir [4] examined climate variables’ short- and long-term impacts on Asia’s agricultural output from 1980 to 2016. It was shown that annual precipitation had a negligible influence on agricultural production in the short term but became beneficial in the long run; on the other hand, the yearly temperature had a detrimental effect on agricultural productivity in the long run.

In the context of India, Bhardwaj et al. [42] observed that the lowest temperature had a beneficial impact on wheat and rice, but a higher temperature had an adverse effect on both crops. Devkota and Paija [46] evaluated climatic factors’ short-run and long-run impact on rice yield in Nepal from 1971 to 2014. They found that rainfall benefits rice output, but minimum and maximum temperatures had a detrimental but statistically negligible effect on rice yield. In lower-middle-income countries, Kumar et al. [47] examined climate change’s impact on cereal production from 1971 to 2016. The study concluded that the adverse effects of temperature on cereal production could seriously affect food security. Conversely, rainfall positively affects cereal production. 

Abbas [43] investigated the impact of climatic factors on the productivity of ten main crops in Pakistan from 2000 to 2019. The study found that climate change has a considerable detrimental impact on the yield of certain crops. Rayamajhee et al. [48] examined the long-term effects of climate change on Nepalese rice production. The findings of this study reveal that extreme rainfall patterns and a rise in average temperatures are significant threats to rice production. Abbas et al. [49] evaluated the influence of climate change on Pakistani food security. According to the research findings, the average temperature has a negative and considerable impact on food production in all parts of Punjab. In the country’s northern region, precipitation negatively affects food production. Kumar et al. [50] found that rainfall has a favorable short-term influence on rice production but an adverse long-term impact; on the other hand, the temperature has an adverse short-term effect on rice productivity. Based on the previous studies, we tested the following hypotheses:

**H1.**  *Technical progress positively affects food crops production in Sichuan province*.

**H2.**  *Climate change negatively affects food crops production in Sichuan province*.

**Table 1:** Summary of empirical studies on the impact of technical progress on grain crops production.

References	Country	Time	Model	Crop	Fertilizers	Mechanization
Ali et al. [38]	Pakistan	1989–2015	ARDL	Wheat + Rice	+/* (wheat)	Not-sig
Ali et al. [39]	Pakistan	1989–2015	ARDL	Sugarcane	+/*	−/*
Chandio et al. [3]	China	1982–2014	KPSS, ADF, PP, ARDL	Agricultural output	+/*	NA
Chandio et al. [29]	Nepal	1990–2016	ARDL	Rice	+/*	NA
Chandio et al. [30]	Nepal	1983–2016	ARDL, VECM, IRF, VD	Maize	+/*	NA
Chandio et al. [31]	Pakistan	1977–2014	ARDL, DOLS, FMOLS, CCR	Cereal production	+/*	NA
Gul et al. [33]	Pakistan	1985–2016	ARDL	Major food crops	+/*	NA
Gul et al. [34]	Pakistan	1970–2018	ARDL, FMOLS, CCR, VECM	Rice	+/*	−/*
Rehman et al. [36]	Pakistan	1978–2015	P–P, ADF	Agricultural output	+/*	NA
Pickson et al. [51]	China	1998–2017	PMG	Rice	+/*	NA
He et al. [2]	China	1978–2018	ARDL	Cereal production	NA	+/*
Abbas [43]	Pakistan	2000–2019	FMOLS, PMG, DOLS	Major crops	+/*	NA

Note: ARDL: Auto Regressive Distributed Lag, KPSS: Kwiatkowski, Phillips, P–P: Phillips–Perron, ADF: Augmented Dickey Fuller, VECM: Vector Error Correction Model, IRF: Impulse Response Function, VD: Variance Decomposition, DOLS: Dynamic Ordinary Least Square, FMOLS: Fully Modified Ordinary Least Square, CCR: Canonical Cointegration Regression, ECM: Error Correction Model, IMFs: Impulse Response Functions, VARD: Variance Decomposition, PMG: Pooled mean group, NA: Not applicable; +: positive; −: negative; *: Significant.

**Table 2:** Summary of empirical studies on the impact of climatic factors on grain crops production.

References	Country	Time	Model	Crop	Temperature	Rainfall
Chandio et al. [32]	Thailand	1969–2016	ARDL, VECM, VARD	Rice	−/*	NA
Gul et al. [33]	Pakistan	1985–2016	ARDL	Major food crops	−/*	+/*
Gul et al. [34]	Pakistan	1970–2018	ARDL, FMOLS, VECM	Rice	−/*	NA
Chandio et al. [35]	Pakistan	1968–2014	ARDL, FMOLS, CCR	Rice	+/*	NA
Bhardwaj et al. [42]	India	1981–2017	FMOLS, DOLS, PMG	Rice + Wheat	−/*	+/*
Devkota & Paija [46]	Nepal	1971–2014	ARDL	Rice	−/*	+/*
Kumar et al. [47]	Selected countries	1971–2016	FGLS, FMOLS	Cereal production	−/*	+/*
Pickson et al. [51]	China	1990–2013	ARDL	Cereal production	−/*	Not-sig
Abbas [43]	Pakistan	2000–2019	FMOLS, PMG, DOLS	Major crops	−/*	NA
Rayamajhee et al. [48]	Nepal	2003–2010	SFM	Rice	−/*	−/*
Abbas et al. [49]	Pakistan	1979–2020	ARDL, ADF, PP	Wheat	−/*	−/*
Kumar et al. [50]	India	1982–2016	ARDL, FMOLS, CCR	Rice	−/*	+/*

Note: ARDL: Auto Regressive Distributed Lag, KPSS: Kwiatkowski, Phillips, Schmidt and Shin, ADF: Augmented Dickey–Fuller, PP: Phillips—Perron, DOLS: Dynamic Ordinary Least Square, FMOLS: Fully Modified Ordinary Least Square, VECM: Vector Error Correction Model, IRF: Impulse Response Function, VD: Variance Decomposition, ECM: Error Correction Model, IMFs: Impulse Response Functions, VARD: Variance Decomposition, JJC: Johansen and Juselius cointegration, SFM: Stochastic frontier model, CCR: Canonical Cointegration Regression, PMG: Pooled Mean Group, FGLS: Feasible Generalized Least Square, NA: Not applicable; +: positive; −: negative; *: Significant.

## 3. Data and Methodology

**Figure 3:**
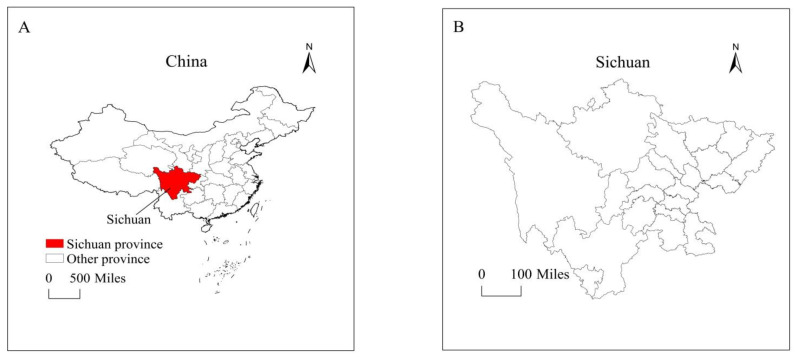
Map of the study area.

**Figure 4:**
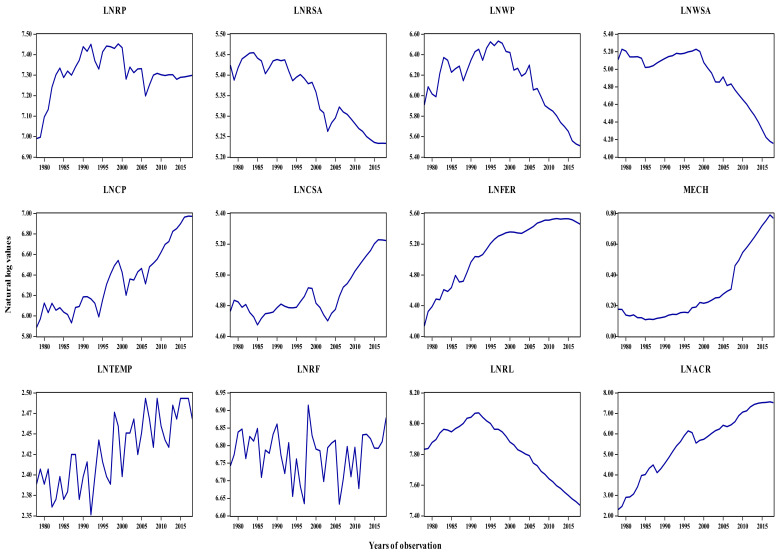
LNRP, LNRSA, LNWP, LNWSA, LNMP, LNMSA, LNFER, LNTEMP, LNRF, LNACR, and LNRL denote the natural log of rice production, rice sown area, wheat production, wheat sown area, maize production, maize sown area, fertilizers used, temperature, rainfall, agricultural credit, and rural labor, while MECH shows mechanical farming rate.

This study examined the impact of technological progress and meteorological factors on major food crop production in Sichuan province, China (see Figure 3). In order to accomplish the core objective of this study, the annual time-series data-set spanning between 1980 and 2018 was accessed from the Sichuan Statistical Yearbook and China Rural Statistical Yearbook [20]. We considered the following variables for the estimation: major food crop production, which included rice production (10,000 tons per hectare), wheat production (10,000 tons per hectare), and maize production (10,000 tons per hectare). Technical factors include fertilizers used (10,000 tons per hectare) and mechanical farming rate (%). Further, this study considers climatological factors such as mean annual temperature (Celsius) and mean annual rainfall (mm) and also includes other vital determinants such as rice sown area (10,000 hectares), wheat planted area (10,000 hectares), maize sown area (10,000 hectares), agricultural credit (100 million RMB), and agrarian labor (10,000 persons). The trend of the studied variables is shown in Figure 4.

Previous studies used several research techniques to estimate the time series and the panel data-set. However, following existing literature [52,53], the current study uses the generalized method of moments (GMM) technique to assess the impact of technological progress and climatological factors on major food crop production. As in this study, we use annual time-series data and apply the GMM. The GMM estimator helps us resolve the possible endogeneity issues in our data-set. This approach (GMM) provides more robust and reliable outcomes than non-instrumental methodologies [54]; however, further instruments added in this method are found to reduce the sample size of the time-series as well as panel data-set [55]. The GMM is one of the most suitable methods in the nexus of climatic variables and agriculture production [52,53]. To achieve our main objective, we have taken the primary form of major food crops production which can be written as: 

*Model I:* The impact of technological progress and meteorological factors on rice production

(1) $$Rice\;production=f\left(Fer,\;Mech,\;Temp,\;Rf,\;Rsa,\;Acr,\;Rl\right)$$

*Model II:* The impact of technological progress and meteorological factors on wheat production

(2) $$Wheat\;production=f\left(Fer,\;Mech,\;Temp,\;Rf,\;Wsa,\;Acr,\;Rl\right)$$

*Model III:* The impact of technological progress and meteorological factors on maize production

(3) $$Maize\;production=f\left(Fer,\;Mech,\;Temp,\;Rf,\;Msa,\;Acr,\;Rl\right)$$

Using the GMM, we have re-formulated the Equations (1)–(3) into natural logarithm as:

(4) $$ln{\left(Rice\right)}_{t}=\alpha_{0}+\Upsilon_{1}ln{\left(Rice\right)}_{t-1}+\Upsilon_{2}ln{\left(Fer\right)}_{t}+\Upsilon_{3}{\left(Mech\right)}_{t}+\Upsilon_{4}ln{\left(Temp\right)}_{t}+\Upsilon_{5}ln{\left(Rf\right)}_{t}+\Upsilon_{6}ln{\left(Rsa\right)}_{t}+\Upsilon_{7}ln{\left(Acr\right)}_{t}+\Upsilon_{8}Ln{\left(Rl\right)}_{t}+\varepsilon_{t}\;$$

(5) $$ln{\left(Wheat\right)}_{t}=\alpha_{0}+\Upsilon_{1}ln{\left(Wheat\right)}_{t-1}+\Upsilon_{2}ln{\left(Fer\right)}_{t}+\Upsilon_{3}{\left(Mech\right)}_{t}+\Upsilon_{4}ln{\left(Temp\right)}_{t}+\Upsilon_{5}ln{\left(Rf\right)}_{t}+\Upsilon_{6}ln{\left(Wsa\right)}_{t}+\Upsilon_{7}ln{\left(Acr\right)}_{t}+\Upsilon_{8}ln{\left(Rl\right)}_{t}+\varepsilon_{t}$$

(6) $$ln{\left(Maize\right)}_{t}=\alpha_{0}+\Upsilon_{1}ln{\left(Maize\right)}_{t-1}+\Upsilon_{2}ln{\left(Fer\right)}_{t}+\Upsilon_{3}{\left(Mech\right)}_{t}+\Upsilon_{4}ln{\left(Temp\right)}_{t}+\Upsilon_{5}ln{\left(Rf\right)}_{t}+\Upsilon_{6}ln{\left(Msa\right)}_{t}+\Upsilon_{7}ln{\left(Acr\right)}_{t}+\Upsilon_{8}ln{\left(Rl\right)}_{t}+\varepsilon_{t}$$

where *Rice* = rice production, *Wheat* = wheat production, *Maize* = maize production, *Fer* = fertilizers used, *Mech* = mechanization, *Temp* = temperature, *Rf* = rainfall, *Rsa*, *Wsa*, and *Msa* = sown area of major food crops (i.e., rice, wheat, and maize), *Acr* = agricultural credit, *Rl* = rural labor, *t* = time period (1980 to 2018), *ln* = for natural logarithm, and $\varepsilon_{t}$ = the error term.

## 4. Results and Discussions

**Figure 5:**
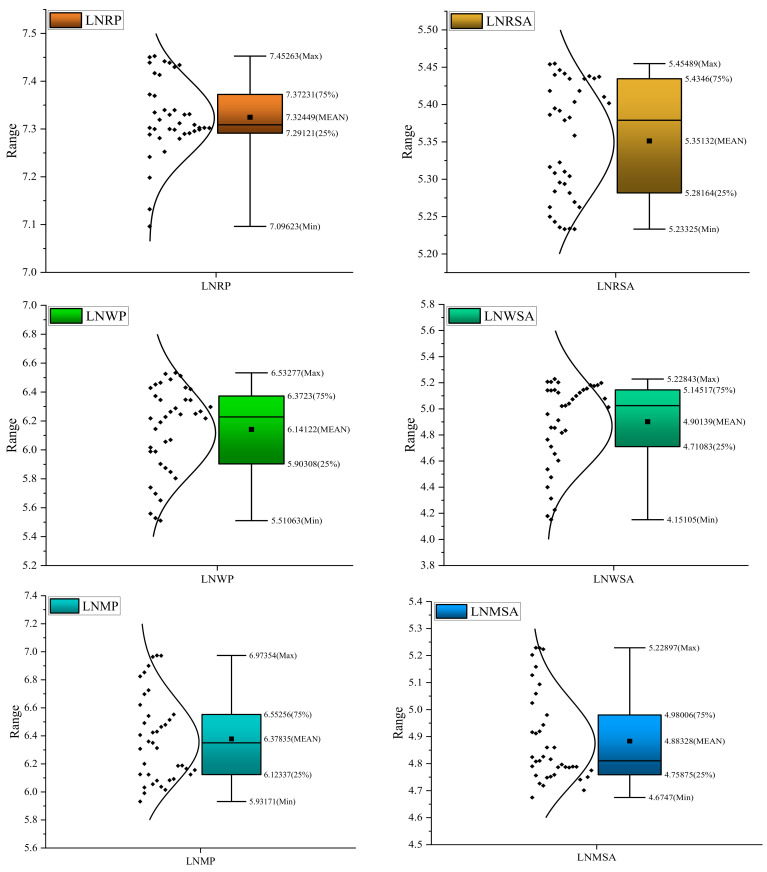

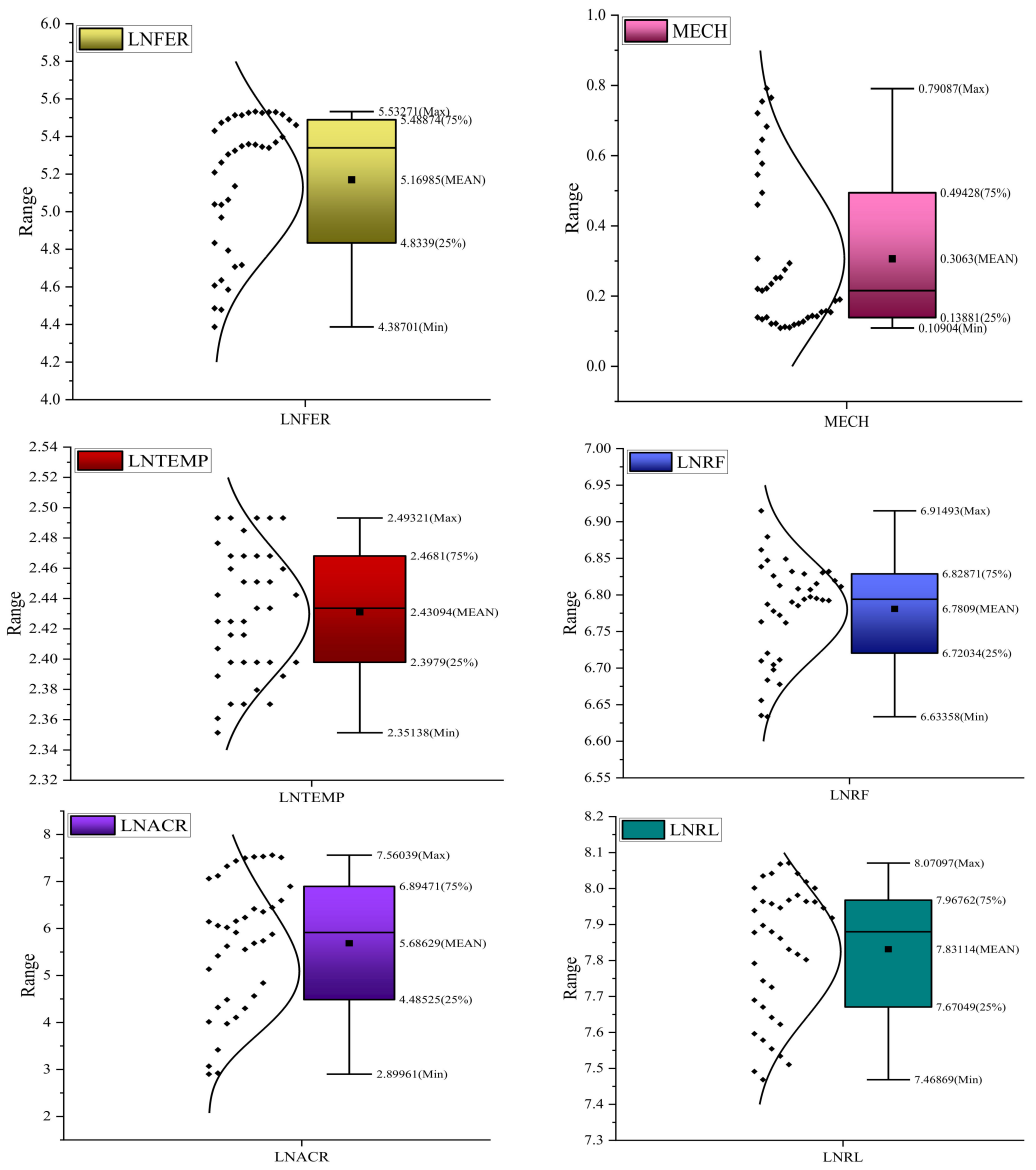
Summary of descriptive statistics in Box plots.

Table 3 provides the summary of the variables that were studied. The estimated statistics of all considered variables reveal that primary food crop production (i.e., rice, wheat, and maize) exhibits +ve mean values, surpassing their standard deviations. Further, this study observed the highest (7.831) and lowest mean value (0.306) in rural labor and mechanization. Except for rice production, all the underlying variables have a Kurtosis value smaller than three. In addition, other variables are generally distributed as per the +ve sign and −ve sign of Skewness. Figure 5 show a summary of descriptive statistics.

Agricultural production and climatic variability are interrelated in various ways because continuous climate change is the prime cause of biotic and abiotic stresses that adversely affect crop production globally [56]. In China, rice (*Oryza sativa*) and wheat (*Triticum aestivum* L.) are produced mainly and consumed as the main staple foods by its teeming population. Table 4 shows the effect of technical progress and meteorological factors on rice output in Sichuan province from 1980 to 2018. Based on the empirical findings of Model (1), at the 1% significance level, increased fertilizer consumption is related to higher rice yield. This result corroborated the assertion of Chandio et al. [29] and Pickson et al. [37], who concluded that fertilizer utilization positively contributed to rice yields in the case of Nepal, Pakistan, and China, correspondingly. Similarly, mechanization has a positive and significant effect on the rice yield of Sichuan province at the 1% level, hence approving the Hypothesis *(H1)*. This result agrees with the findings of Zhou and Ma [57], who used data from 29 provinces in China and showed the positive nexus between mechanization and farming productivity. 

Furthermore, in the case of Nigeria, Takeshima et al. [58], and in the context of sub-Saharan Africa, South Asia, and Latin America, Van Loon et al. [59] revealed that mechanized farming had higher crop productivity. The temperature, in contrast, is significantly and negatively related to rice production at the 10% level, thus endorsing the Hypothesis *(H2)*. This means higher average temperature brings about lower rice output in Sichuan province. This result is congruent with Bhardwaj et al. [42] and Gul et al. [34], who reported that increased temperature had a detrimental effect on rice cultivation in India, Thailand, and Pakistan. 

**Figure 6:**
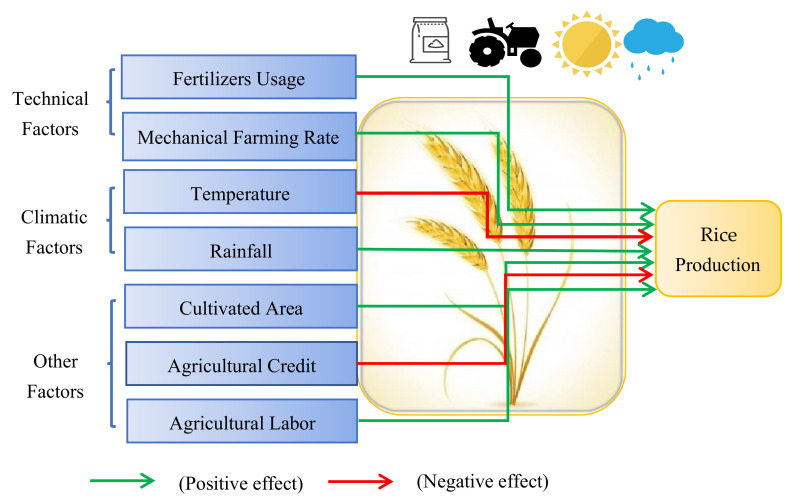

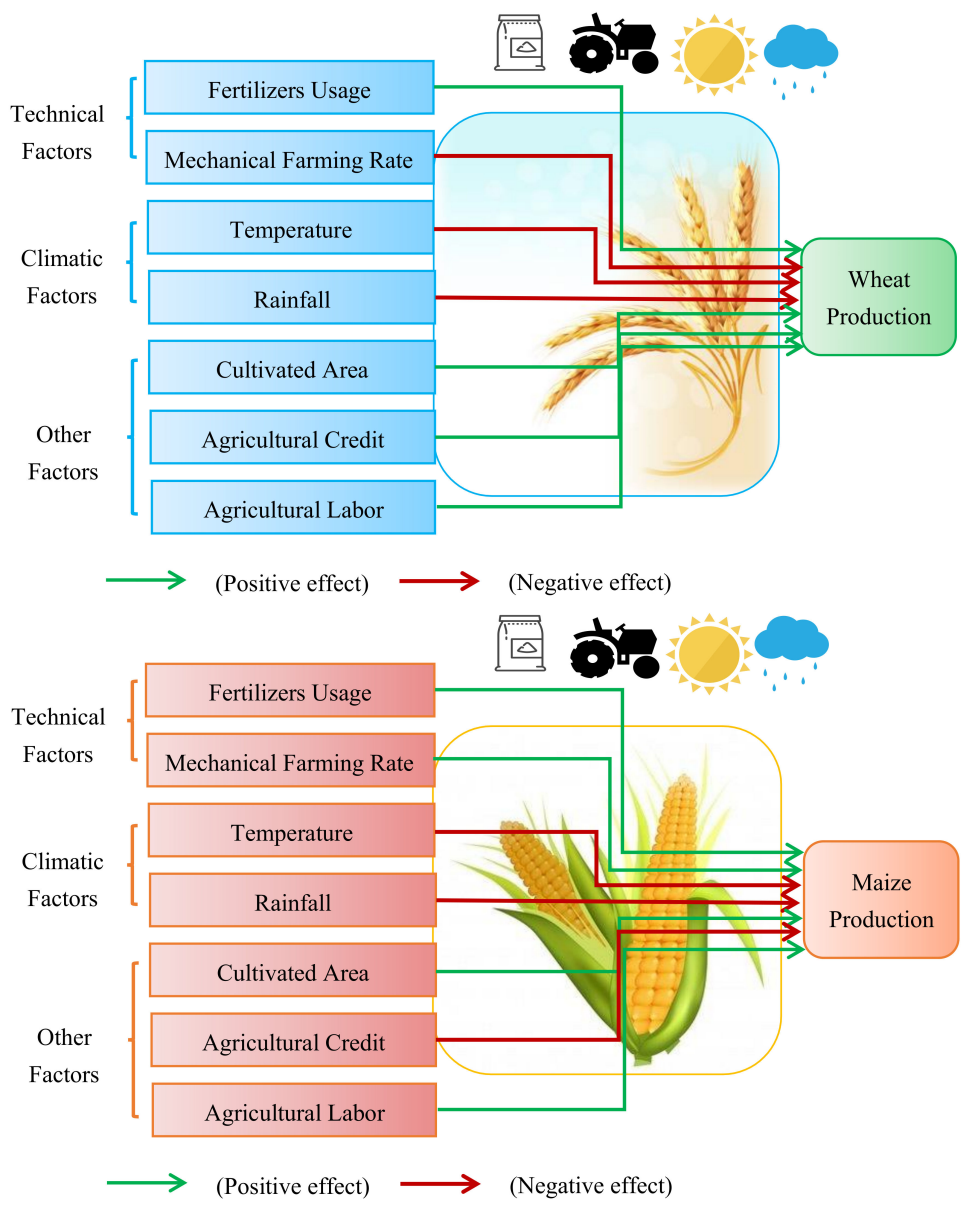
Key findings for Models 1, 2, and 3.

This finding also backed up the claim of Wang et al. [11], Li et al. [60], and Pickson et al. [37], who found that climate factors adversely and significantly affected rice production in China. As rice is one of the primary crops in China [51], the negative effect of global warming on rice crops suggests that the food security in China is currently threatened. Rainfall, however, did not affect the rice production of Sichuan province during the investigated period. Thus, consistent with the result of Ali et al. [36], there is no association between precipitation and rice yield. At the 1% significance level, the empirical finding of Model (1) likewise suggests a positive relationship between rural labor and rice production. The findings from the GMM method for Model 1 are displayed in Figure 6.

**Table 4:** The effect of technical progress and climate change on rice productivity (Model 1).

Variables	Coefficient	Std. Error	t-Statistic	Prob.
_Cons	−2.881607	1.784180	−1.615087	0.1168
lnRP (−1)	0.246357	0.113660	2.167498	0.0383
lnFER	0.259965	0.085917	3.025749	0.0051
MECH	0.623438	0.166159	3.752058	0.0008
lnTEMP	−0.486761	0.277146	−1.756333	0.0892
lnRF	0.038773	0.114849	0.337600	0.7380
lnRSA	0.129937	0.412785	0.314782	0.7551
lnACR	−0.034713	0.025730	−1.349087	0.1874
lnRL	0.931849	0.226162	4.120273	0.0003
R^2^	0.773248		Adjusted R^2^	0.712781
D-W stat	1.414508		J-stat	2.143243

Note: Dependent variable is ln(RP). Instrumental list is comprised of the lag value of independent variables. ln denotes the natural logarithm. FER and MECH denote the technical factors, TEMP and RF denote the climatic factors, RSA, ACR, and RL refer other determinants and RP is rice production.

Sustainable and healthy food production is essential to fulfilling the domestic food demand of the rapidly growing population. Hence, the farming sector needs to optimize the technical progress to achieve this goal and improve green economic growth. Winter wheat is commonly cultivated in several Chinese provinces. However, some provinces grow winter and spring wheat based on suitable climatic conditions and achieve higher yields. Although Sichuan province also produces winter wheat, the production is lower as compared to other wheat-producing provinces.

Regarding the effects of farming techniques and weather conditions on wheat production in Sichuan province, the empirical results in Table 5 demonstrate that fertilizer utilization positively affects wheat production at the 5% significance level; consequently, we confirm the Hypothesis *(H1)* of our study. This result affirms Ali et al.’s [38] findings that fertilizer usage significantly increases wheat output. Temperature, in contrast, adversely impacts wheat production at the 1% significance level, implying the unwanted effect of climate change on wheat production in Sichuan province; as a result, we confirm the Hypothesis *(H2)* of our study. Since wheat is the second major crop in China that contributes significantly to the national food security [61], this result indicates a severe issue of climate change that dramatically affects food security in China. This result supports Huang et al. [9], Ali et al. [38], and Bhardwaj et al. [42], who figure out the adverse relationships between temperature and wheat yields in China, Pakistan, and India, respectively. Additionally, Table 5 demonstrates that wheat production is significantly affected by the rural labor in Sichuan province. The outcomes from the GMM technique for Model 2 are also demonstrated in Figure 6. 

Presently, maize (*Zea mays* L.) is widely grown as a food crop around the globe, and it covers 193.7 million hectares of planting area with an annual production of 1147.6 million tons [62]. In Sichuan, the maize crop is also extensively grown and considerably contributes to the livestock industry. Table 6 exhibits the significant and positive influence of fertilizer usage and mechanical farming rate on maize yields at the 1% and 5% significance levels; hence, it is noteworthy to confirm the Hypothesis *(H1)* of this investigation. The results signify a vital role of technical progress in maize production in Sichuan province. These results are similar to the conclusions of Chandio et al. [30] and Zhou et al. [63] that usage of fertilizer and farm machinery positively impacts maize production in Nepal and China correspondingly. However, the climate changes with temperature and rainfall factors negatively affected maize yields in Sichuan province during the investigated period; consequently, we endorse the Hypothesis *(H2)* of this investigation. This finding is congruent with maize’s feature that it can withstand moderate to high temperatures [64,65]. The result on the temperature-maize production nexus is negative but non-significant, while in previous findings, the temperature is significantly and negatively associated with rice and wheat crop yields, suggesting that maize could be an alternative to rice and wheat under ongoing climate change. In Table 6, agricultural credit negatively and significantly decreases maize production, implying the ineffectiveness of using credit in developing maize crops. Finally, Figure 6 shows the overall results of the GMM approach for models 1, 2, and 3.

## 5. Conclusions

Being a significant grain-producing province in western China, Sichuan supplies substantial cereal outputs to the country. This region, however, has experienced a marked change in climatic conditions that exhibit a higher average temperature and less precipitation. Therefore, this study scrutinized the impacts of technical progress (fertilizer use and mechanization) and climate change (temperature and rainfall) on major food crops’ output to reveal the determinants of food crop outputs in Sichuan province. 

Overall, farming techniques and meteorological factors heterogeneously influence major crop productions in Sichuan province. Specifically, our empirical results reveal the positive nexus between fertilizer utilization and outputs of all major crops (rice, wheat, and corn). Regarding mechanization, only rice and corn yields are significantly and positively impacted. In terms of climatic factors, increased average temperature greatly diminishes rice and wheat yields, suggesting the threat of climate change to the food security of Sichuan province and China. Rainfall, nevertheless, is unlikely to have a significant effect on any crop production in Sichuan province. In addition, rural labor contributes positively to major food crops in Sichuan province.

### 5.1. Policy Implications

According to the empirical findings, our study proposes several recommendations to enhance the current major crop production in Sichuan province and ensure food security in China. First, the effectiveness of using fertilizer is evident in Sichuan province’s primary food crop production. Hence, applying quality fertilizers with recommended doses is critical to achieving higher rice, wheat, and corn outputs in the region. Second, mechanization also plays an essential role in increasing food crop production. Therefore, the use of technology in food crop cultivation should be vigorously promoted.

Finally, due to the current level of global warming and its effect on food production in the country, China’s government should consider revising its strategies and policies to reduce the impact of climate change on food crop production and increase the adaptive ability of farmers. In particular, there should be a provincial program to improve the quality of cultivation areas with a better irrigation system. The farmers also need to be supported to select cultivars suitable for higher temperatures and adopt climate-resilient agricultural technology that brings about long-term food security for the region and the whole country.

### 5.2. Limitation and Future Research Path

Achieving sustainable food production and green economic growth is a prime objective of the Chinese government. Hence, the current study used annual time-series data from 1980 to 2018 and applied the GMM approach to examine the impacts of climatic changes (via an average yearly temperature and an average annual rainfall) on the top three main staple food crops, rice, wheat, and maize production of Sichuan Province-China. The key findings revealed that climate change adversely influences primary food crop production. Moreover, this study suggests that academicians and researchers may consider the seasonal minimum and maximum temperature and precipitation level to assess the climate change impacts on food crop yield and incorporate the influential role of crop-wise chemical fertilizers use, agricultural machinery, and public policy support programs in major food crops producing provinces of China by using a panel database.

## Figures and Tables

**Table 3:** Descriptive statistics.

Variables	Obs.	Mean	Std. Dev	Min	Max	Skewness	Kurtosis
lnRP	39	7.324	0.080	7.096	7.452	−0.485	3.766
lnRSA	39	5.351	0.077	5.233	5.454	−0.182	1.492
lnWP	39	6.141	0.299	5.510	6.532	−0.651	2.323
lnWSA	39	4.901	0.322	4.151	5.228	−1.025	2.851
lnMP	39	6.378	0.308	5.931	6.973	0.532	2.152
lnMSA	39	4.883	0.164	4.674	5.228	0.965	2.625
lnFER	39	5.169	0.363	4.387	5.532	−0.783	2.199
MECH	39	0.306	0.225	0.109	0.790	1.013	2.474
lnTEMP	39	2.430	0.042	2.351	2.493	−0.094	1.866
lnRF	39	6.780	0.067	6.633	6.914	−0.591	2.734
lnACR	39	5.686	1.402	2.899	7.560	−0.430	2.174
lnRL	39	7.831	0.183	7.468	8.070	−0.557	1.991

Note: lnRP, lnRSA, lnWP, lnWSA, lnMP, lnMSA, lnFER, lnTEMP, lnRF, lnACR, and lnRL denote the natural logarithm of rice production, sown area of rice, wheat production, sown area of wheat, maize production, sown area of maize, fertilizers used, temperature, rainfall, agricultural credit, and rural labor, while MECH means mechanization.

**Table 5:** The impact of technological progress and climate change on wheat production (Model 2).

Variables	Coefficient	Std. Error	t-Statistic	Prob.
_Cons	1.641766	4.455109	0.368513	0.7151
lnWP (−1)	0.459326	0.189632	2.422193	0.0217
lnFER	0.200000	0.068682	2.911969	0.0067
MECH	−0.293055	0.210186	−1.394266	0.1735
lnTEMP	−1.892169	0.544067	−3.477822	0.0016
lnRF	−0.004890	0.645698	−0.007573	0.9940
lnWSA	0.021784	0.163566	0.133180	0.8949
lnACR	0.023577	0.026385	0.893580	0.3787
lnRL	0.654596	0.100562	6.509367	0.0000
R^2^	0.946107		Adjusted R^2^	0.931736
D-W stat	1.714645		J-stat	3.248512

Note: Dependent variable is ln(WP). FER and MECH denote the technical factors, TEMP and RF denote the climate change factors, WSA, ACR, and RL refer other determinants and WP is wheat production. The instrumental list is comprised of the lag value of independent variables.

**Table 6:** The impact of technical progress and climate change on corn production (Model 3).

Variables	Coefficient	Std. Error	t-Statistic	Prob.
_Cons	3.138381	4.347365	0.721904	0.4759
lnMP (−1)	−0.840622	0.435557	−1.929995	0.0631
lnFER	1.668068	0.584661	2.853051	0.0078
MECH	3.136348	1.388329	2.259080	0.0313
lnTEMP	−0.396964	0.538677	−0.736924	0.4669
lnRF	−0.206295	0.372776	−0.553403	0.5841
lnMSA	0.122215	0.573120	0.213246	0.8326
lnACR	−0.413497	0.165381	−2.500264	0.0181
lnRL	0.406761	0.582054	0.698837	0.4900
R^2^	0.700403		Adjusted R^2^	0.620511
D-W stat	0.566122		J-stat	7.324322

Note: Dependent variable is ln(MP). FER and MECH denote the technical factors, TEMP and RF denote the changing climate factors, MSA, ACR, and RL refer other determinants and MP is production of maize. The instrumental list contains the lagged value of independent variables.

## Data Availability

The data will be available on request.
